# Trigger factor assisted self-assembly of canine parvovirus VP2 protein into virus-like particles in *Escherichia coli* with high immunogenicity

**DOI:** 10.1186/s12985-018-1013-8

**Published:** 2018-06-19

**Authors:** Liangliang Nan, Yunchao Liu, Pengchao Ji, Hua Feng, Chen Chen, Juan Wang, Dongmin Liu, Yinglei Cui, Yanwei Wang, Yafei Li, Enmin Zhou, Gaiping Zhang

**Affiliations:** 1 College of Veterinary Medicine, Northwest A&F University, Yangling, 712100 China; 2 Henan Provincial Key Laboratory of Animal Immunology, Henan Academy of Agricultural Sciences, Zhengzhou, 450002 China; 3 College of Animal Science and Veterinary Medicine, Henan Agricultural University, Zhengzhou, 450002 China; 4 Henan Zhongze Biological Engineering Co., Ltd., Zhengzhou, 450019 China

**Keywords:** Canine parvovirus, *Escherichia coli*, Immunogenicity, Trigger factor 16, Virus-like particle

## Abstract

Canine parvovirus (CPV) has been considered to be an important pathogen, which can cause acute infectious disease in canids. Although current vaccines are effective in preventing CPV infection, safety problems still remain unsolved. In this study, a subunit vaccine against CPV based on virus-like particles (VLPs) with good safety and immunogenicity is reported. Soluble CPV VP2 protein was produced by co-expression of chaperone trigger factor (Tf16) in *Escherichia coli* (*E.coli*), and assembled into CPV VLPs which could be affected by NaCl and pH. At 250 mM NaCl pH 8.0, the VLPs co-expressed with Tf16 had similar size (25 nm) and shape with the authentic virus capsid under the transmission electron microscopy (TEM), which is also in accordance with the dynamic light scattering (DLS) data. Immunization with these particles could induce high-titer hemagglutination inhibition (1:12288) and neutralizing antibodies (1:6144) in guinea pigs. Splenic cells of them could secrete IFN-γ and IL-4 after stimulation by CPV. Thus, the VLPs produced by the new approach with high yield and immunogenicity could be a potential candidate for CPV vaccine.

## Introduction

Canine Parvovirus (CPV) disease is a highly contagious infectious disease caused by canine parvovirus, which is manifested as severe hemorrhagic enteritis in dogs of all ages and myocarditis in puppies [1]. Since it was first discovered in 1978, domestic dogs and some wild animals have been facing a huge risk because of the high morbidity and mortality throughout the world [2]. CPV belongs to genus *Protoparvovirus* within family *Parvoviridae* and has the ability to agglutinate pig erythrocytes [3]. It is a 25 nm diameter particle consisting of three structural proteins, VP1, VP2 and VP3. As the main component of viral capsid, VP2 protein can spontaneously assemble into VLPs and exhibit good immunogenicity [4].

CPV-2 was the original virus strain in dogs that spread worldwide, and soon gave rise to three antigenic variants with mutation sites in VP2 protein [5]. CPV-2a contained 5 amino acid substitutions (M87L, I101T, A300G, D305Y, and V555I) and is the predominant variant in Asia; CPV-2b had a single additional substitution (N426D) and an I555V reversion; CPV-2c featuring N426E and S297A is the predominant variant in Europe and Latin America [6–8].

Vaccination plays an important role in the prevention of this disease. Attenuated CPV-2 vaccines are effective and widely used, but large scale production of them is usually expensive and laborious. Although the attenuated CPV-2 vaccine strains are stable in limited passages in the dog, a series of CPV-2-like strains were identified and deduced to evolve from the vaccine strains [8]. Inactivated CPV vaccines are not recommended for routine use. They are less effective and take much longer to induce an immune response when compared with attenuated vaccines [9]. Thus, it is significant to develop alternative vaccines, such as virus-like particles (VLPs) based vaccine. VLPs are composed of viral structural proteins but lack viral genome. They could be recognized easily by the immune system and show dramatic effectiveness because of the present of viral antigens in a conformation like virus [10]. It was reported that CPV VLPs with good immunogenicity could be produced by the eukaryocyte system [4]. However, this method is complicated and costs a lot.

Prokaryotic expression system has been used extensively for recombinant proteins production in laboratory and industrial scale due to its simplicity, rapid growth rate and relatively low cost. Actually, it is not uncommon that overexpressed recombinant proteins fail to reach a correct conformation and are prone to associate with each other to form insoluble aggregates [11]. To cope with this situation, an increase in the intracellular concentration of molecular chaperones may be a useful strategy. Tigger factor (TF) is one of the most important molecular chaperones and the only prokaryotic chaperone associated with the ribosome [12]. It has a cleft-like concave binding pocket for potential substrates to assist proper folding of them in the crowded cellular environment [13]. Co-expression of TF has successfully enhanced yield and biological activity of some recombinant proteins in *Escherichia coli (E.coli*), such as bovine carbonic anhydrase II [14].

In this study, we obtained high-yield soluble VP2 protein by co-expression with chaperone Tf16 in *E.coli*. Under the optimum condition (250 mM NaCl pH 8.0), the VLPs assembled by the VP2 protein were used to immunize guinea pigs. The immunological tests suggested that this kind of VLPs has high immunogenicity and may be a promising candidate vaccine against CPV.

## Materials and methods

### Cells and virus

Feline kidney F81 cells were cultured in Dulbecco’s modified Eagle’s medium (DMEM; Hyclone), which was supplemented with 100 U/ml of penicillin, 100 mg/ml of streptomycin and 10% fetal bovine serum (FBS; Gibco) at 37 °C in a 5% CO_2_ incubator.

The virus was isolated from feces of an infected puppy as described previously [15]. DNA was extracted from the infected F81 cells by TIANamp Virus DNA/RNA Kit (Takara, China). The VP2 gene was amplified by PCR and sequenced (Sangon, China). To examine the titration, F81 cells were seeded into 96-well plates. At 60% confluency, cells were overlaid with serially 10-fold diluted supernatant of CPV-infected cells for 1 h at 37 °C. Then the supernatant was changed to medium supplemented with 3% FBS. After 72 h incubation at 37 °C, the cytopathic effect (CPE) was assessed by immunoperoxidase monolayer assay (IPMA). The CPV was used for all the immunological tests in this study.

### Plasmid construction and expression

The sequence of VP2 protein was optimized based on CC8601 strain (GenBank accession number GU569948). The complete VP2 gene was amplified by a pair of primers (Songon, China). The forward primer was 5’-GAGGATCC (*Bam*HI) ATGAGCGATGGTGCAGT-3′, and the reverse primer was 5’-GATCTCGAG (*Xho*I) TTAGTACAGTTTGCGCGG-3′. The VP2 gene was digested by the restriction endonucleases *Bam*HI and *Xho*I, and sub-cloned into pET30a (pET30a-VP2). Then the recombinant plasmid was transformed into *E. coli* BL21 (DE3) competent cells harboring pTf16, which were prepared according to the manufacturer’s protocol (TaKaRa, China).

The colony, positive for both VP2 protein and Tf16, were cultured into LB medium containing 2 mg/ml L-Arabinose, 0.1 mM IPTG, 0.1 mg/ml chloramphenicol and kanamycin. After 14 h induction at 25 °C, the cells were harvested and lysed by sonication in buffer A (400 mM NaCl, 50 mM Tris, pH 8.0) on ice. The supernatant and debris were collected by centrifugation at 10000 g for 20 min and analyzed by SDS-PAGE.

### Purification of CPV VP2 protein

The VP2 protein with an N-terminal His tag was purified by Ni-NTA affinity chromatography (Merck, Germany). Briefly, the clarified supernatant was loaded on the Ni-NTA column at a flow rate of 1 ml/min for 3 times. The column was washed with buffer B (400 mM NaCl, 50 mM Tris, 30 mM imidazole, pH 8.0) until no protein was detected by CBB staining (TIANGEN, China). The recombinant VP2 protein was thoroughly eluted with buffer C (400 mM NaCl, 50 mM Tris, 150 mM imidazole, pH 8.0) and analyzed by SDS-PAGE and Western blot.

### VLPs assembly condition and characterization

The eluted VP2 protein was dialyzed against 50 mM Tris with different concentrations of NaCl (150 mM, 250 mM, 400 mM) and pH (pH 7.0, pH 8.0) at 4 °C for 16 h. To analyze the assembly condition and appearances of VLPs, the collected protein was detected by dynamic light scattering (DLS) and transmission electron microscopy (TEM).

### Guinea pigs immunization with CPV VLPs

Thirty female guinea pigs housed under pathogen-free conditions were randomly divided into five groups (*n* = 5). Group A, B and C were intramuscularly immunized with 10 μg, 30 μg and 50 μg CPV VLPs mixed with Montanide™ ISA 201 VG (Seppic, France), respectively; group D was inoculated with 100 μl commercial live-attenuated vaccine as a positive control; group E was injected with 100 μl PBS as a negative control. Booster immunization with the same doses of VLPs was performed at 21 dpi (days post inoculation). Blood samples were collected from the forelimb veil at 0, 14, 21, 28, 35 and 42 dpi.

### Anti-CPV antibody detection

All serum samples from guinea pigs were inactivated at 56 °C for 30 min and diluted in a two-fold series starting at 1:30. After incubation with 100 TCID_50_ of CPV at 37 °C for 2 h, the virus/serum mixtures were added to 96-well plates containing F81 cells at 37 °C and 5% CO_2_. After three days, the cytopathic effect (CPE) was assessed by immunoperoxidase monolayer assay (IPMA). The highest dilution of sera showing complete inhibition of CPE was taken as the neutralization titer.

Hemagglutination inhibition (HI) assay was performed as described previously [3]. Briefly, serially diluted serum samples were mixed with 4 hemagglutination (HA) units of virus antigen. The mixtures were then incubated with freshly prepared 0.8% pig erythrocytes in 96-well V-shaped microtiter plates. HI antibody titers were expressed as the reciprocal of the final dilution of serum which completely inhibited hemagglutination.

### Analysis of cytokine production

Spleens of guinea pigs from each group were removed under sterile conditions at 42 dpi. Cells were washed twice with RPMI 1640 containing 10% FBS and filtered by Cell Strainers (Falcon, USA). Red blood cells (RBCs) in the cell suspension were lysed using RBC lysis buffer (20 mM Tris, 160 mM NH_4_Cl, pH 7.4) for 6 min at room temperature. Then spleen lymphocytes were seeded in 96-well plates and stimulated by CPV. After 48 h’ incubation at 37 °C, cell culture supernatants were tested using commercial IL-4 and IFN-γ ELISA kits (Bogoo, China) according to the manufacturer’s instructions.

### Statistical analyses

All values were tested for significance by one-way analysis of variance (ANOVA) using GraphPad Prism version 5.00 (GraphPad Software, San Diego, CA, USA). The significance level was set at 0.05 (*p* < 0.05).

## Results

### Expression and purification of VP2 protein

**Fig. 1:**
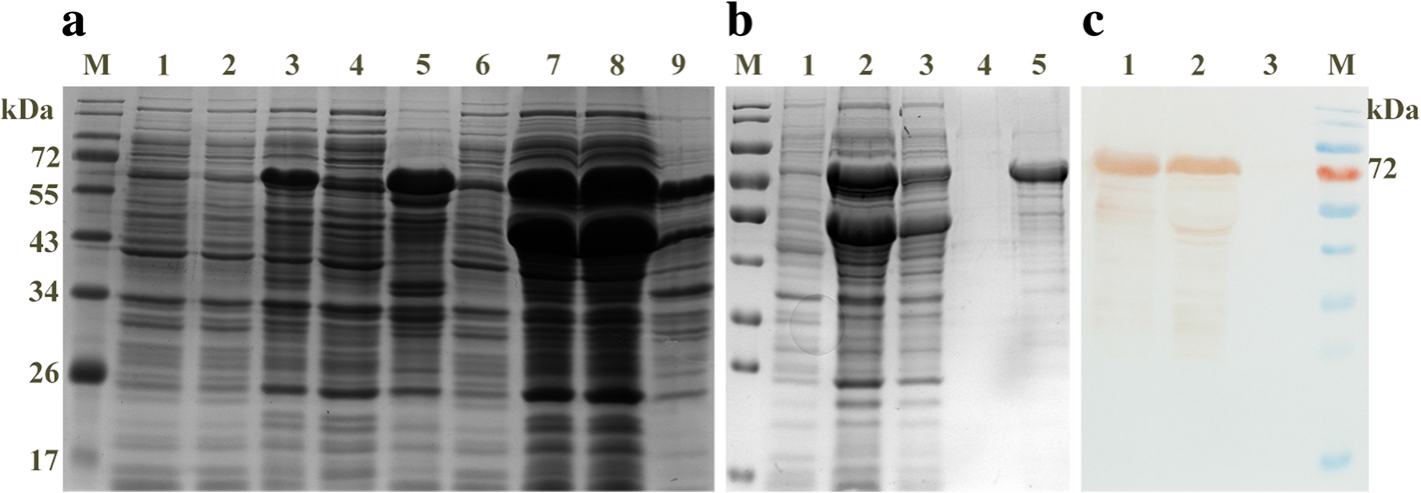
Expression and purification of CPV VP2 protein co-expressed with Tf16. CPV VP2 protein were analyzed by 12% SDS-PAGE and Western blot using an anti-CPV polyclonal antibody **a** SDS-PAGE analysis of the VP2 protein expression. Lane M, protein molecular weight (MW) markers; Lane 1, pET30a vector control; Lane 2, pET30a-VP2 cells before induction of IPTG; Lane 3, induced pET30a-VP2 cells; Lane 4, induced pET30a-VP2 cell lysate; Lane 5, inclusion body of induced pET30a-VP2 cells; Lane 6, pET30a-VP2-Tf16 cells before induction of IPTG and L-Arabinose; Lane 7, induced pET30a-VP2-Tf16 cells; Lane 8, induced pET30a-VP2-Tf16 cell lysate; Lane 9, inclusion body of induced pET30a-VP2-Tf16 cells. **b** SDS-PAGE analysis of purification of VP2 protein co-expressed with chaperone Tf16. Lane 1, cells before induction; Lane 2, induced cells lysate; Lane 3, flow buffer; Lane 4, wash buffer; Lane 5, purified VP2 protein. **c** Western-blot analysis of purified VP2 protein with an anti-CPV polyclonal antibody. Lane1, purified VP2 protein; Lane 2, induced cell lysate; Lane 3, cells before induction. The MW of VP2 protein is approximately 70 kDa. The Tf16 is approximately 56 kDa

The soluble VP2 protein was expressed by co-transformation with pTf16 and confirmed through SDS-PAGE and Western blot. The result showed that a 70 kDa protein was produced corresponding to the predicted molecular weight of His-VP2 protein (Fig. 1a). The yield of soluble VP2 protein was improved after co-expression with Tf16 (56 kDa), and the purity of it could be about 90% after purification by Ni-NTA affinity chromatography (Fig. 1b). Western blot analysis further confirmed that the purified 70 kDa protein is the target protein by using an anti-CPV polyclonal antibody (Five Star, China) (Fig. 1c).

### Assembly condition and characterization of VLPs

**Fig. 2:**
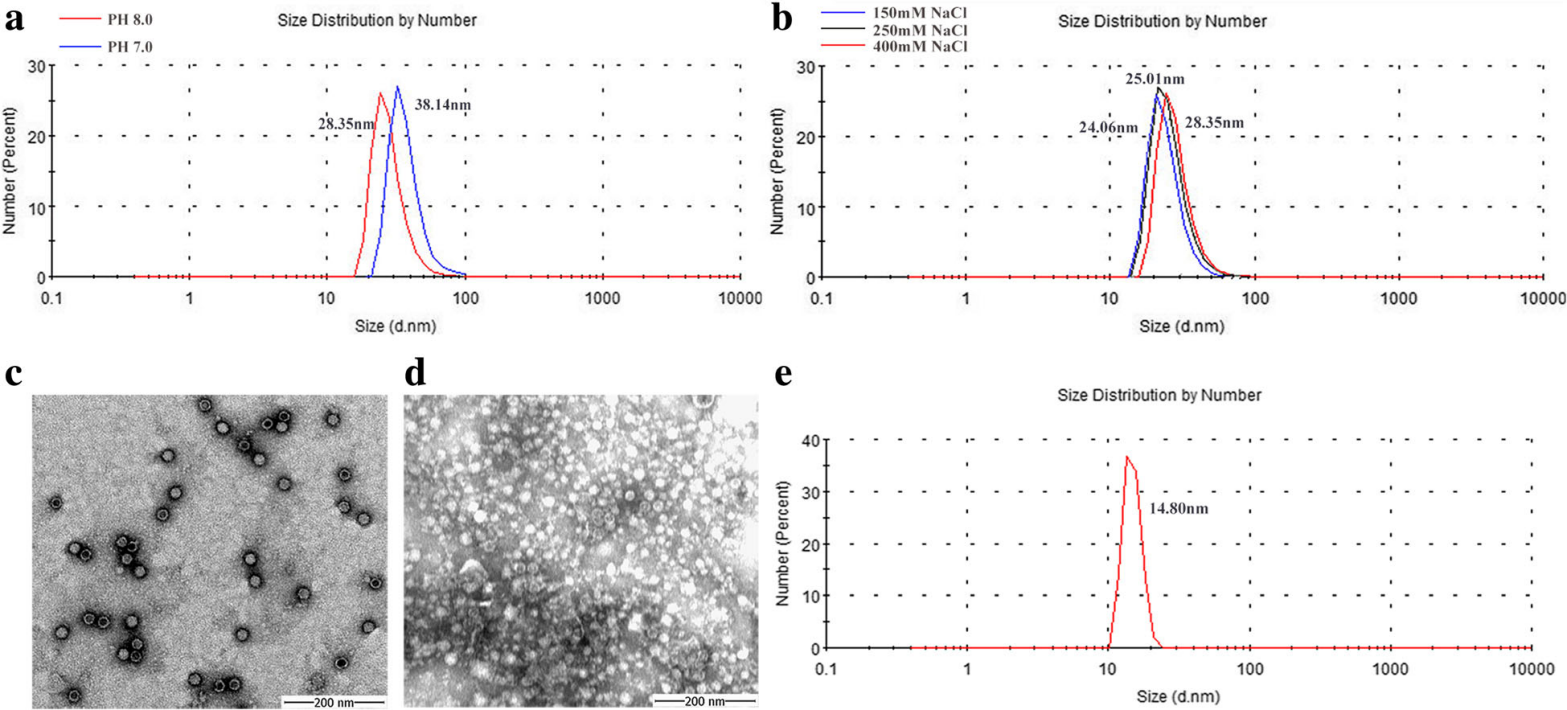
Assembly and characterization of CPV VLPs under different conditions. **a** DLS results of VLPs assembled at pH 7.0 and 8.0. **b** DLS results of VLPs assembled in different concentrations of NaCl (150, 250, 400 mM). **c** TEM result of VLPs co-expressed with Tf16. Bar = 200 nm **d** TEM result of VLPs without co-expression of Tf16. Bar = 200 nm **e** DLS result of VLPs without co-expression of Tf16

To understand the effect of pH and NaCl on the assembly of CPV VLPs, the purified protein were analyzed by DLS. As demonstrated in Fig. 2a, there are more VLPs with same size as CPV (25 nm) at pH 8.0 than that at pH 7.0. Thus, the optimal salt concentration was further analyzed at pH 8.0. DLS data showed that the size of recombinant VLPs changed within a narrow range and became larger as the increase of NaCl concentration. The highest amount of particles, with a dimension of approximately 25 nm, was obtained at 250 mM NaCl pH 8.0 (Fig. 2b). They also have similar shape with the authentic virus capsid under TEM (Fig. 2c). However, relatively small particles (14.80 nm) were formed without co-expression of molecular chaperone Tf16 and showed unclear edges under TEM (Fig. 2d and e).

### Genotyping of isolated CPV

DNA sequencing was performed for the amplified CPV VP2 gene. Nucleotide sequence comparisons to current CPV revealed that the virus belongs to CPV-2a strain and shares 100% VP2 identity with strain SD-14-5 (GenBank accession number KR611515).

### Antibody response in guinea pigs

**Fig. 3:**
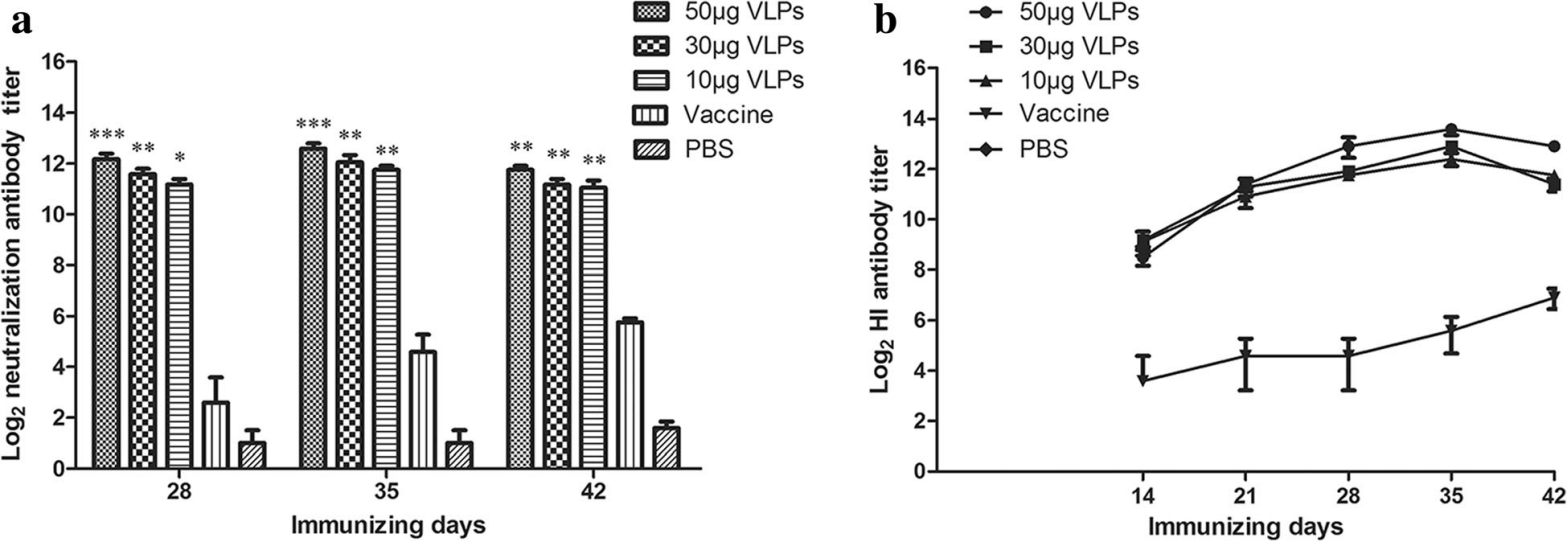
Anti-CPV antibody responses in guinea pigs. Groups of guinea pigs (*n* = 5) were immunized with 50 μg, 30 μg and 10 μg of CPV VLPs, commercial CPV vaccine and PBS. Serum samples were collected once a week until 42 dpi for the detection of neutralization and HI antibody titers. **a** Neutralization antibody titers of guinea pigs. **b** HI antibody titers of guinea pigs. The values are expressed as means ± SEM. The statistical significance of antibody titer differences between vaccinated and PBS control guinea pigs were analyzed by one-way ANOVA statistical analysis (*p < 0.05, **p < 0.01, and ***p < 0.001)

To test whether CPV VLPs could induce specific humoral immune responses, guinea pigs were divided into five groups and immunized with different doses of CPV VLPs (10 μg, 30 μg, 50 μg), 100 μl commercial CPV vaccine and PBS. As shown in Fig. 3a, neutralizing antibody titers of 50 μg group increased dramatically and then reached to the highest (1:6144) at 35 dpi. Meanwhile, high-titer HI antibodies were also induced strongly in 50 μg group and peaked (1:12288) at 35 dpi (Fig. 3b). The humoral immune responses of VLPs groups changed in a dose-dependent manner and shared similarities in the growth trend. The antibody titers of them were consistently significantly higher (*p* < 0.05) than PBS group after the second immunization.

### Assays of cytokines

**Fig. 4:**
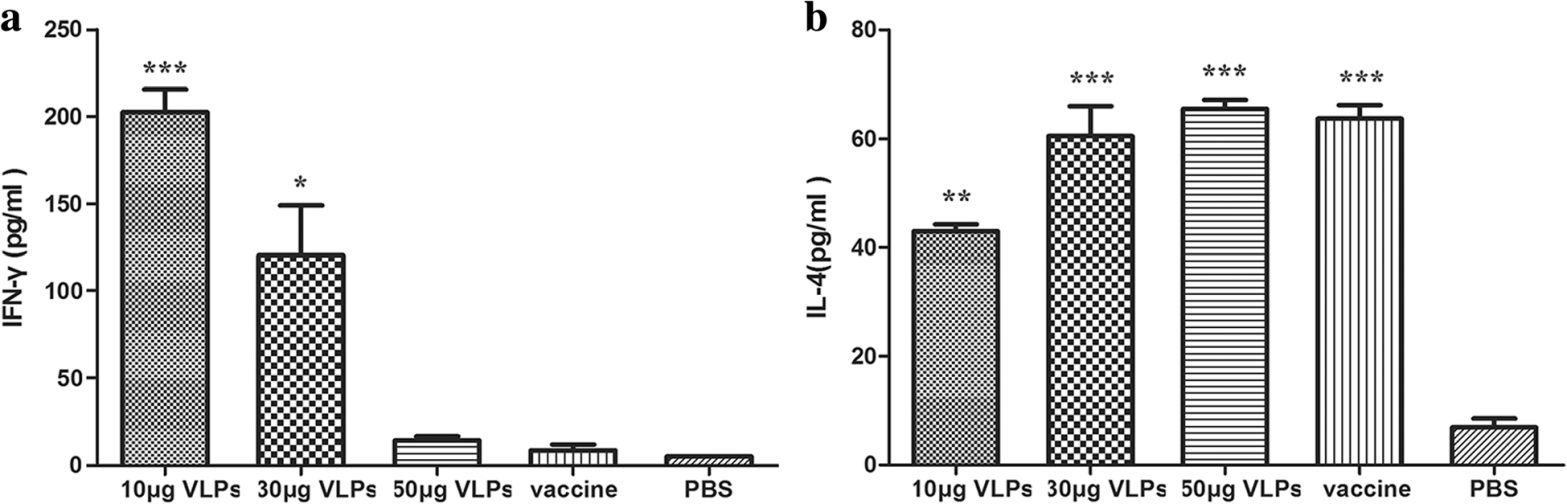
Analysis of cytokine production secreted from the spleen lymphocytes of guinea pigs. Spleens of guinea pigs from each group were removed at 42 dpi. The culture supernatants of cells stimulated by CPV were collected after 72 h and detected by ELISA kits. **a** the concentration of guinea pig IFN-γ in each group. **b** the concentration of guinea pig IL-4 in each group. The values are expressed as means ± SEM. The statistical significance of cytokine concentration differences between vaccinated and PBS control guinea pigs were analyzed by one-way ANOVA statistical analysis (*p < 0.05, **p < 0.01, and ***p < 0.001)

IFN-γ and IL-4 from the splenocytes of guinea pigs were detected by ELISA. As Fig. 4a shown, the amount of IFN-γ was significantly higher in 10 μg (*p* < 0.001) and 30 μg (*p *< 0.05) immunized groups than the control group. No significant difference was found between the commercial CPV vaccine and PBS group. The IL-4 content of VLPs vaccinated groups changed in a dose-dependent manner. The VLPs and commercial vaccine groups showed significantly higher (*p* < 0.01) IL-4 production compared to the control group (Fig. 4b).

The stimulated T lymphocytes produced a much higher concentration of IFN-γ compared to IL-4 in both the 10 μg and 30 μg VLPs groups, but the situation was opposite in the commercial vaccine group. As shown in Fig. 4b, the mean amount of IL-4 secreted in the guinea pigs vaccinated with 10 μg and 30 μg of the VLPs was about 42 and 60 pg/ml, respectively. And the mean amount of IFN-γ secreted in the same groups was about 202 and 120 pg/ml (Fig. 4a). However, 64 pg/ml IL-4 and 9 pg/ml IFN-γ were secreted in the commercial vaccine group (Fig. 4a and b).

## Discussion

The most important enteric virus infecting canids is CPV. Although injection of attenuated vaccine is an effective way to control this disease currently, it may bring some safety concerns for immunized dogs [9]. The need for developing alternative vaccines, such as VLP-based vaccine, would be very urgent. It mimics virus particles, presenting considerable antigenic epitopes which can stimulate a diverse set of immune responses [16]. In this study, we produced CPV VLPs by co-expression with Tf16 in *E.coli*. These particles could induce strong immune responses in guinea pigs.

It has been predicted that VP2 protein with high molecular weight and weak hydrophilicity, is more prone to accumulate as inclusion bodies in *E.coli* [17]. Some methods failed to achieve in VP2 soluble expression [18, 19] and correct refolding of the protein. In this study, the proportion of the VP2 protein in supernatant increased after the involvement of Tf16. One reason for its high solubility may be that Tf16 has the ability to prevent VP2 nascent chains from misfolding and aggregation after being translated by the ribosome [20, 21]. This is a quick and easy method for producing high yield protein, because we can also remove Tf16 conveniently from the supernatant (Fig. 1b). A previous study reported that high yield soluble VP2 protein could be successfully produced with small ubiquitin-like modifier (SUMO) fusion motif in *E.coli* [17], but the SUMO-VP2 protein needs cleavage and re-purification, which may affect the yield of mass production.

The stability of viral protein and the formation process of VLPs are susceptible to salt concentration and pH [22]. These effects could be detected by DLS and TEM, which are the common approaches for VLPs size measurement and could verify the assembled condition of particles [23]. In the current study, pH-induced assembly of CPV VLPs was observed by DLS, which is similar with a previous study about human parvovirus B19 VLPs [24]. The effect indicated the involvement of ionizable groups at the interfaces between CPV capsid proteins. In addition, salt can stabilize protein by influencing ionic interaction on its surface [25], so we added different concentration of NaCl in the buffer. At pH 8.0, the size of VLPs changed within a narrow range as the increase of NaCl concentration. This suggested that ionic strength (150–400 mM NaCl) may make less difference to VLPs assembly in our study. Although the CPV VLPs assembled efficiency was unknown, it was estimated that the VLPs could be formed with similar size and shape as CPV at 250 mM NaCl pH 8.0. Interestingly, a relatively small particle (14.80 nm) was formed without Tf16 co-expression and showed unclear edges under TEM, which may result from the incorrect folding and insufficient amount of VP2 protein.

To evaluate the immunogenicity of CPV VLPs, anti-CPV antibodies were tested in the immunized guinea pigs. In VLPs immunized groups, HI antibody titers increased rapidly after the second immunization may because of the stimulated memory B cells. It is remarkable that the maximum titers of HI antibody and neutralization antibody were 1:12288 and 1:6144 respectively at 35 dpi in 50 μg VLPs group. A powerful response was also observed in 10 μg group, despite the lower immune dose compared to the previous study [17, 26]. It indicated that the antibodies could easily bind to antigens on the surface of CPV to prevent entering cells and agglutinating pig erythrocytes. Such good immunogenicity suggested that the VP2 protein expressed in *E. coli* could be fold correctly with natural bioactivity. Strong humoral immune responses may also result from the appropriate adjuvant (Montanide™ ISA 201 VG) which is robust, stable, with low viscosity and easy to inject, compared to traditional oil emulsions. In addition, the low level of HI antibodies (1:192) and neutralization antibodies (1:60) induced by the commercial CPV vaccine may be related to the absence of a host animal. Here, we just regard the commercial vaccine as a positive control and a challenge test will be performed in dogs to evaluate the immune protection in the future.

It has been well documented by previous studies that CPV VLPs could induce proliferation of CD4^+^ and CD8^+^ T cells in mice [17, 26]. Furthermore, T-helper type 1 (Th1) and T-helper type2 (Th2) cells are two subsets of CD4^+^ T cells which related to cellular and humoral adaptive immunity, respectively [27]. In this study, IFN-γ (Th1-type cytokine) and IL-4 (Th2-type cytokine) were measured after CPV stimulation to evaluate the Th1 and Th2 responses. In the 10 μg and 30 μg VLPs groups, stimulated T lymphocytes produced a much higher concentration of IFN-γ compared to IL-4, but in the commercial vaccine group, the situation was opposite. The results indicated that the VLPs may mainly stimulate Th1 response compared with the commercial vaccine in guinea pigs. The strong Th1 type-specific immune response attained partly by interaction of VLPs with DCs can also be induced by several other kinds of VLP-based vaccines, such as the VLPs from human papilloma virus, influenza virus, hepatitis B virus and human immunodeficiency virus-1 [28–31]. However, the secretion of IFN-γ decreased as the VLPs dose increased, and even no significant difference was found between 50 μg VLPs and PBS groups. This trend was consistent with a previous study [32] and requires further experiments to understand the details.

In summary, the CPV VP2 protein co-expressed with Tf16 in *E.coli* could assemble into VLPs similar with natural CPV virions in size and shape. The VLPs could stimulate strong immune responses in guinea pigs against CPV. Thus, the Tf16 co-expression system provides a new approach for producing CPV VLPs with high yield and efficiency. With the following improvements, a CPV VLP-based vaccine may be developed successfully in the future.

## Abbreviations

CPV: Canine parvovirus
VLPs: Virus-like particles
TF: Trigger factor
*E.coli*: *Escherichia coli*
TEM: Transmission electron microscopy
DLS: Dynamic light scattering
CPE: Cytopathic effect
IPMA: Immunoperoxidase monolayer assay
HI: Hemagglutination inhibition
HA: Hemagglutination
RBCs: Red blood cells
ANOVA: Analysis of variance
SUMO: Small ubiquitin-like modifier
